# Association between women's empowerment and fertility preferences in Ghana

**DOI:** 10.1093/inthealth/ihae043

**Published:** 2024-06-08

**Authors:** Louis Kobina Dadzie, Hilda Yengnone, James Boadu Frimpong, Ebenezer Agbaglo, Abdul-Aziz Seidu, Bright Opoku Ahinkorah

**Affiliations:** Department of Population and Health, University of Cape Coast, Cape Coast, Ghana; Medical Social Welfare, Cape Coast Teaching Hospital, Cape Coast, Ghana; School of Justice, Faculty of Creative Industries, Education and Social Justice, Queensland University of Technology, Brisbane, Queensland, Australia; Kumasi Centre for Research in Tropical Medicine, Kumasi, Ghana; Department of Health, Physical Education and Recreation, University of Cape Coast, Cape Coast, Ghana; Department of Kinesiology, New Mexico State University, Las Cruces, NM, USA; Department of English, University of Cape Coast, Cape Coast, Ghana; Centre for Gender and Advocacy, Takoradi Technical University, Takoradi, Ghana; College of Public Health, Medical and Veterinary Sciences, James Cook University, Townsville, Queensland, QLD 4811, Australia; School of Clinical Medicine, University of New South Wales, Sydney, New South Wales, Australia; REMS Consultancy Services, Sekondi Takoradi, Western Region, Ghana

**Keywords:** children, fertility preference, Ghana, married women, women’s empowerment

## Abstract

As Ghana has embraced the concept of women’s empowerment as a vital tool for sustainable development, it has become crucial to evaluate the role that women’s empowerment plays in the fertility preferences of married and cohabiting women in the country. The study's objective was to examine the association between women's empowerment, the ideal number of children and women's ability to have their desired number of children. This cross-sectional study used data from the 2014 Ghana Demographic and Health Survey. Both Poisson and binary logistic regression analyses were carried out. Women who had justification for wife-beating (incidence rate ratio [IRR] 0.98 [95% confidence interval {CI} 0.96 to 0.99]) and those who were autonomous (IRR 0.94 [95% CI 0.93 to 0.95]) had lower rates of having the ideal number of children. Moreover, women who had justification for wife-beating (adjusted odds ratio [aOR] 1.25 [95% CI 1.13 to 1.39]) and those who were involved in decision-making (aOR 1.31 [95% CI 1.19 to 1.44]) had higher odds of having the ability to have the desired number of children. However, autonomous women (aOR 0.78 [95% CI 0.71 to 0.86]) had lower odds of having the ability to have the desired number of children. Significant associations were found between women's empowerment (women's attitude towards justification for wife-beating, autonomy), an ideal number of children and the ability to have the desired number of children. These findings present target areas for policies and interventions aimed at determining Ghanaian women's fertility preferences and empowering them.

## Introduction

The empowerment of women is a means to achieve sustainable development worldwide, with emphasis on promoting women's sense of worth, their ability to make their own choices, their right to have the power to control their own lives within and outside the home and their right to influence the direction of social change.^1^ According to Reshi and Sudha,^2^ women's empowerment entails equipping women with the essential resources and opportunities to assert their rights, engage in decision-making and wield authority over their own lives. Efforts targeted at promoting gender equality and women’s empowerment saw the passing of Millennium Development Goal 3 (MDG3) in 2000,^3^ but the uneven achievements and expiration of the MDGs by 2015 led to the Sustainable Development Goals (SDGs) in June 2015 to carry the momentum generated by the MDGs beyond 2015.^4^ Ghana adopted the MDGs in 2000 and implemented policies that equip and include women in sustainable development, such as increasing women's participation in government by 40%, which saw only 13.1% of women being represented in the legislature by 2020. SDG5 seeks to achieve gender equality and women’s and girls’ empowerment by the year 2030 and has resulted in a narrowed gap of gender inequality. However, Ghana lags behind other sub-Saharan African countries like Namibia and Rwanda by 20%.^5^

Women's empowerment plays a key role in women's control of their fertility and provides them with the opportunity to make decisions about reproduction, such as contraception and deciding the number, timing and spacing of their children.^6^ The United Nations, through the World Population Prospects, has reported a decrease in fertility, with the main contributor being a decrease in ideal family size, a component of fertility preference.^7^ Evidence suggests a causal relationship between women's empowerment and fertility; lower fertility leads to greater women's empowerment and vice versa; fertility is negatively associated with women's education and employment due to exposure to modern ideas and values that promote individualism.^8^ However, these findings are yet to be investigated in Ghana, where, in past years, there has been a consistent decrease in fertility rates. A 1.32% decrease in 2020 alone places the fertility rate in the country at 3.75 births per woman in 2021. This decrease in fertility preferences is due to the availability of modern contraceptive methods.^9^ Fertility preferences in the country have been associated with socio-economic status, age, marital status, income, parity and education status.^10^

Skills development, education, independent decision-making ability and control over household resources have greatly impacted fertility preferences, with similar trends existing where women's empowerment has resulted in lower fertility, longer birth intervals and lower rates of unintended pregnancy in southern Asia.^9^ A woman can influence and negotiate for safer sex practices when empowered, as she has autonomy in decision-making and is also able to help resolve disagreements over contraception, which might be a potential threat from their partners, as violence has been significantly implicated in fertility preference.^9,11,12^ A study reported that wealthy and highly educated women were more likely to refuse sexual intercourse and to even tell their partners to use condoms, thus gaining control over their fertility preference.^13^ As Ghana has embraced the concept of women’s empowerment as a vital tool for sustainable development, it has become crucial to evaluate the role that women’s empowerment plays in fertility preferences in married and cohabiting women in Ghana.

## Methods

### Source of data

This study used the 2014 Ghana Demographic and Health Survey (GDHS) data. The GDHS is nationally representative and employed a cross-sectional design. The survey comprised four main questionnaires: a household questionnaire, a women’s questionnaire, a men's questionnaire and a biomarker questionnaire. These provide data mainly on socio-economic status, fertility, reproductive health and maternal and child health. For this study, we used the individual recode (women's) data file, with a sample size of 5204 women. Women who were not married were not included in this study and missing data were omitted. Data are available online upon request at https://dhsprogram.com.

### Study area

This study focused on Ghana, a country that lies within the West African region. At the time of the survey, Ghana was divided into 10 administrative regions that consisted of 216 districts to ensure equitable distribution of resources and effective and efficient administration at the local level.^14^ However, Ghana has redemarcated the regions and added 6 more since the previous round, bringing the total to 16 regions with 216 districts, including 145 districts, 109 municipal districts and 6 metropolitan districts.^15^ Several ethnic groups exist within the nation, with the Akans constituting the largest and others including the Ga-Adangbe, Gonja and Dagbani.^15^

### Variables

#### Dependent variables

This study used two dependent variables: the ideal number of children a married woman desires to have and the ability to limit fertility to achieve the stated ideal number. The first dependent variable was deduced from the question, ‘If you could go back to the time you did not have any children and could choose exactly the number of children to have in your whole life, how many would that be?’. A similar question was asked differently for those who do not have living children: ‘If you could choose exactly the number of children to have in your whole life, how many would that be?’. Responses to these questions were both numeric and non-numeric. Based on evidence,^16–18^ we coded non-numeric responses as the average to the rest of the country sample to minimize bias. The other dependent variable (ability to limit fertility to achieve the stated ideal) was binarily created. This was calculated as the number of living children minus the number of desired children (i.e. an ideal number of children). Women with a difference of more than zero were considered as having more children than desired and vice versa.^17,18^

#### Independent variables

The main independent variable for the study was women's empowerment. We adopted the Survey-based Women's Empowerment (SWPER) index, which decomposed empowerment into three dimensions: attitude towards wife-beating, woman's autonomy and involvement in decision-making. Attitude towards wife-beating was composed of five questions that asked whether the partner is justified in beating the wife if the wife goes out without telling the husband, neglects the children, argues with the husband, refuses to have sex with the husband or burns food. Autonomy included responses to questions on the frequency of reading newspapers or magazines, working in the last 12 months, the woman's education, the education difference between the husband and wife, age at cohabitation and age of respondent at the first birth. Decision-making variables were responses to who usually decides on respondents’ healthcare, large household purchases and visits to family or relatives. For recoding of these variables, see Table 1. These were used to generate scores for attitudes towards violence, autonomy and decision-making using principal component analysis. A more detailed methodological approach for generating the scores has been published elsewhere.^19^

**Table 1.** Variables included in the SWPER for Ghana

Variable	Category
**Attitude towards violence**	
Beating justified if wife goes out without telling husband	Yes, −1; don't know, 0; no, 1
if wife neglects the children	
if wife argues with husband	
if wife refuses to have sex with husband	
if wife burns the food	
**Autonomy**	
Frequency of reading newspapers or magazine	Not at all, 0; <once a week, 1; ≥once a week, 2
Respondent worked in last 12 months	No, 0; in the past year, 1; have a job, but on leave last 7 d, 2; currently working, 2
Woman’s education (years of schooling)	Years
Education difference: woman−husband years of schooling	Years
Age difference: woman−husband	Years
Age at first cohabitation	Years
Age of respondent at first birth	Years
**Decision-making**	
Who usually decides on respondent's healthcare	Husband/other alone, −1; joint, 0; respondent alone, 1
Who usually decides on large household purchases	
Who usually decides on visits to family or relatives	

#### Covariates

We included other measures as covariates. These were place of residence, wealth, husband’s/partner's education level, household size, religion and nature of union (polygamous or not). For better presentation, variables such as husband’s/partner's education level, household size and religion were recoded. The husband’s/partner's education level was recoded as no formal education, primary, secondary and higher; the household size, which was previously continuous, was recoded as 1–3, 4–6, 7–9 and 10+; and religion was recoded as no religion, Christian, Islam and traditionalist.

### Data analyses

Characteristics of the categorical variables used for the study were described as frequencies and percentages. Mean values of continuous variables were also provided. We also presented a summary statistics of the various women’s empowerment scores to provide an overview. To assess the association between the first dependent variable (ideal number of children) and women’s empowerment, basic checks were done to investigate the appropriate statistical technique to adopt since the outcome was continuous. As evidenced from a histogram with kernel density, skewness/kurtosis and the Shapiro–Wilk test (see Appendix A), it was clear that the residuals were not bell-shaped, so use of an ordinary least squares regression would not be appropriate. However, statistical results providing the mean (4.93) and variance (4.28) showed that the application of Poisson regression would be appropriate since the variance is not far from the mean. We employed the Poisson regression to determine the association between women’s empowerment and the ideal number of children in two models. Model 1a incorporates the women’s empowerment dimensions against the outcome and model 1b controls for the covariates. The results were graphically presented as an incidence rate ratio (IRR) with confidence intervals (CIs) and in a table (see Appendix B). The second analysis comprised an association between women's empowerment and a dichotomously created outcome (the ability to have the desired number of children). Associations were established using a binary logistic regression. The modelling followed the same approach as the first dependent variable. Results were presented graphically and in tables (see Appendix C) with odds ratios (ORs) and accompanying CIs. All analyses were weighted and adjusted for complex survey design. A p-value <0.05 was set as statistical significance. Stata version 15.0 (StataCorp, College Station, TX, USA) was used to perform all the analyses.

## Results

### Descriptive results

Table 2 shows the descriptive results of the independent variables considered in this study. A total of 5204 women were included in the study. A greater proportion responded that they resided in urban areas (n=2606 [50.1%]) and were in the richest wealth index (n=1229 [23.6%]). Most of the respondents reported that their husband’s or partner’s highest education level was secondary (n=2999 [57.6%]), had a household size of 4–6 (n=2633 [50.6%]), belonged to the Christian religion (n=4007 [77%]), were not in a polygamous union (n=4401 [84.6%]), did not have the ability to have the desired number of children (n=3815 [73.3%]) and the mean ideal number of children was 4.9.

**Table 2.** Descriptive statistics of independent variables (weighted n=5204)

Variable	n	%
Place of residence		
Urban	2606	50.1
Rural	2598	49.9
Wealth index		
Poorest	1000	19.2
Poorer	935	18
Middle	978	18.8
Richer	1063	20.4
Richest	1229	23.6
Husband’s/partner's education level		
None	1064	20.4
Primary	501	9.6
Secondary	2999	57.6
Higher	641	12.3
Household size		
1–3	1412	27.1
4–6	2633	50.6
7–9	879	16.9
≥10	280	5.4
Religion		
None	170	3.3
Christian	4007	77
Islam	893	17.2
Traditional	134	2.6
Polygamous union		
No	4401	84.6
Yes	803	15.4
Ability to have the desired number of children		
No	3815	73.3
Yes	1389	26.7
Ideal number of children, mean		4.9

#### Women’s empowerment scores

Table 3 shows the results of the average scores of women’s empowerment. The study found that the average scores of the women’s empowerment dimensions were 0.27 (standard deviation [SD] 0.87), 0.20 (SD 1.05) and 0.55 (SD 0.87) for attitude towards violence, autonomy and decision-making, respectively.

**Table 3.** Summary statistics of SWPER women’s empowerment dimensions

Statistic	Attitude towards violence	Autonomy	Decision-making
Mean	0.27	0.20	0.55
P50/median	0.75	0.03	0.55
SD	0.87	1.05	0.87
Minimum	−2.14	−2.40	−1.74
Maximum	0.94	5.17	2.64

#### Association between women’s empowerment and the ideal number of children

**Figure 1.**
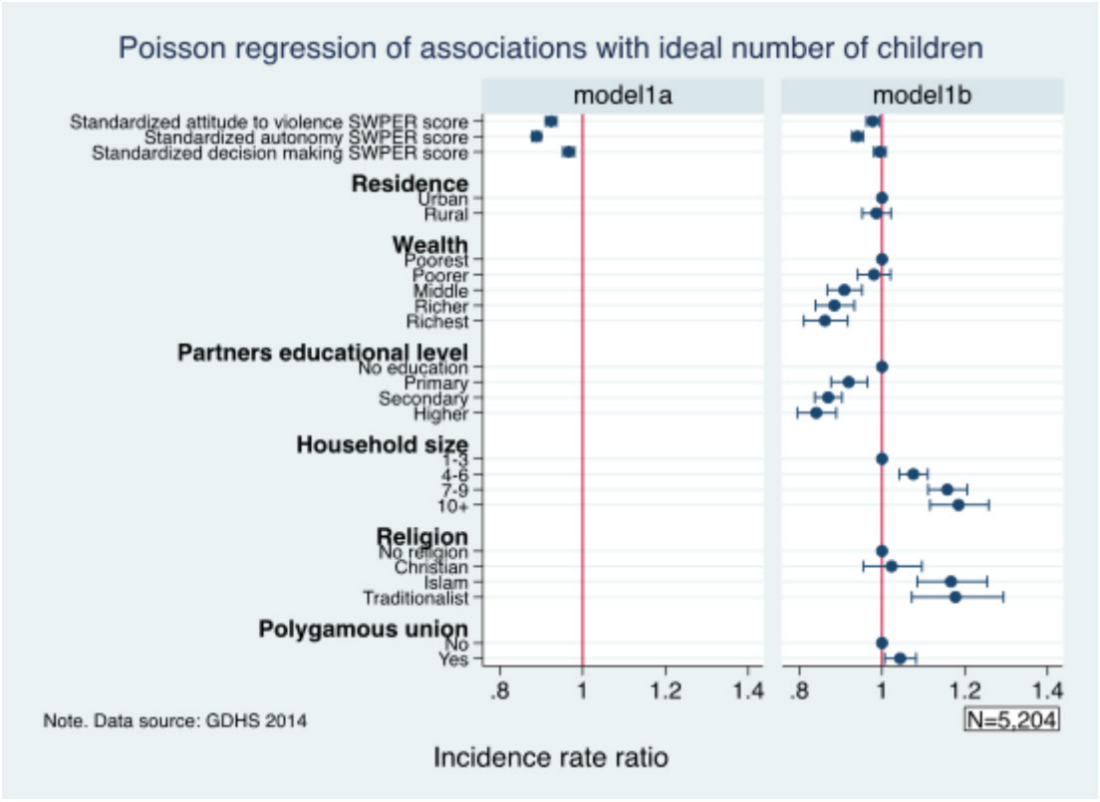
Association between women’s empowerment and the ideal number of children.

Figure 1 presents the results for the association between women's empowerment and desire for more children. The study found that women who justified wife-beating (IRR 0.98 [95% CI 0.96 to 0.99]) and those who were autonomous (IRR 0.94 [95% CI 0.93 to 0.95]) had lower rates of having a desire for more children. The study also found that the richest women (IRR 0.86 [95% CI 0.81 to 0.92]) and those whose husbands had higher education (IRR 0.84 [95% CI 0.80 to 0.89]) had the lowest rates of having the desire for more children. In contrast, women who had a household size of ≥10 (IRR 1.19 [95% CI 1.12 to 1.26]), belonged to the traditional religion (IRR 1.18 [95% CI 1.07 to 1.29]) and were in a polygamous union (IRR 1.04 [95% CI 1.01 to 1.08]) had the highest rates of having the desire for more children.

#### Association between women’s empowerment and ability to have the desired number of children

**Figure 2.**
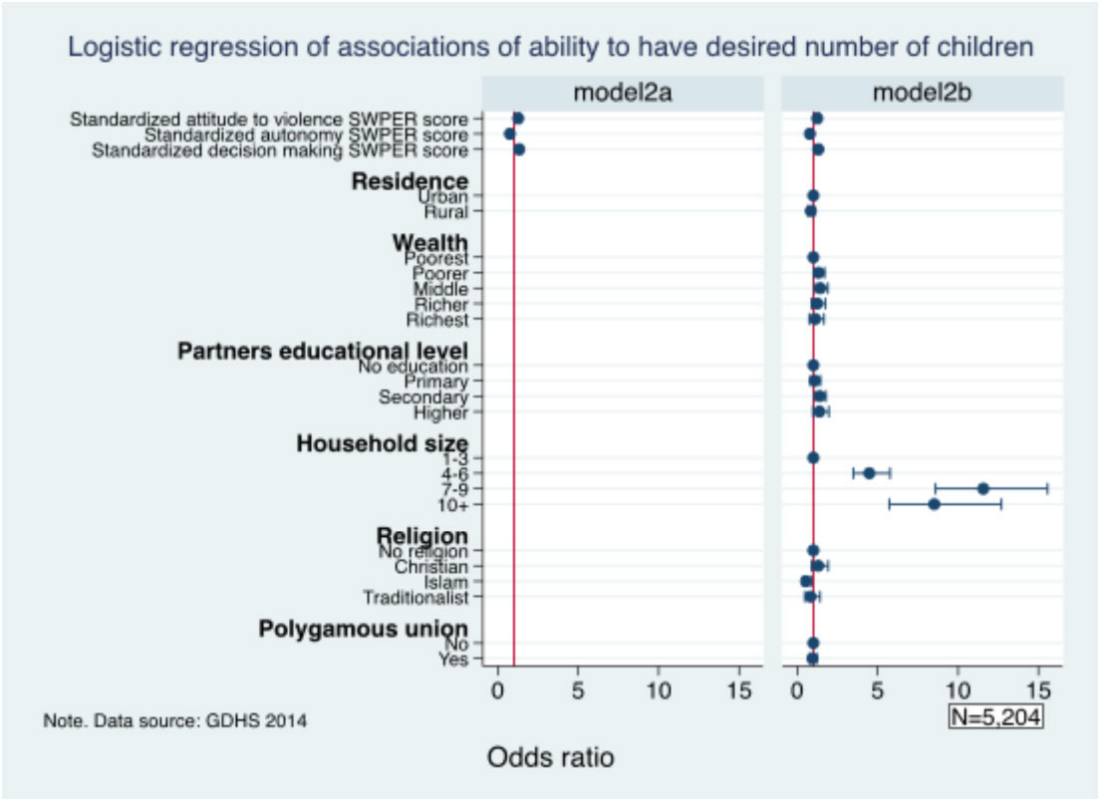
Association between women’s empowerment and the ability to have the desired number of children.

Figure 2 shows the results of the association between women’s empowerment and the ability to have the desired number of children. The study found that women who justified wife-beating (adjusted OR [aOR] 1.25 [95% CI 1.13 to 1.39]) and those who were involved in decision-making (aOR 1.31 [95% CI 1.19 to 1.44]) had higher odds of having the ability to have the desired number of children. However, autonomous women (aOR 0.78 [95% CI 0.71 to 0.86]) had lower odds of having the ability to have the desired number of children. The study also found that women who were in the middle wealth index (aOR 1.43 [95% CI 1.06 to 1.91]), whose husband had a secondary education (aOR 1.40 [95% CI 1.09 to 1.80]) and had a household size of 7–9 (aOR 11.57 [95% CI 8.60 to 15.57]) had the highest odds of having the ability to have the desired number of children. Women who belonged to the Islamic religion (aOR 0.55 [95% CI 0.35 to 0.86[) had lower odds of having the ability to have the desired number of children.

## Discussion

This study assessed women's empowerment and fertility preferences among married and cohabiting women in Ghana. The results show that reproductive preferences are related to several aspects of women's empowerment. Both the ideal number of children and the desired number of children were found to be influenced by additional confounders, including sociodemographic characteristics. In the sections below, we discuss these associations.

The study found that women who justified wife-beating and those who were autonomous had lower odds of having the ideal number of children. This finding echoes the findings of Snow et al.^20^ who reported on the association between gender attitudes and ideal family size in Ethiopia, Rwanda, Tanzania, Uganda and Zambia. The study by Snow et al.^20^ found that women who tolerated wife-beating were likely to have additional children to their husband's ideal number of children. This suggests that male dominance increases the number of children beyond the woman's ideal number. In addition, Khan and Islam^21^ and Tareque et al.^22^ emphasized justification of wife-beating as a barrier to accessing healthcare services, including antenatal care and family planning. A study by Khan and Bari^23^ supports the findings that attitudes towards violence significantly reduces the ideal number of children. This research can assist policymakers in suggesting interventions to eliminate or reduce these practices.

The negative association between women's autonomy and the ideal number of children can be explained in several ways that reflect the complex interplay of social, economic and cultural factors that shape reproductive choices and family planning decisions.^18^ This finding also exposes some limitations in the measures of autonomy. Measuring women's autonomy can be problematic due to the wide array of attitudes and practices the concepts potentially encompass.^24^ In the present study, autonomy was defined to include the frequency of reading newspapers or magazines, working in the last 12 months, the woman's education, the education difference between the husband and wife, age at cohabitation and age of the respondent at the first birth. Some of these dimensions do not necessarily suggest women's autonomy. For example, the fact that a woman frequently reads newspapers does not necessarily mean that she is autonomous and capable of having the ideal number of children.

Andarge et al.^25^ explained that when women have more children, they tend to live for the children, thus tolerating all kinds of violence towards themselves just to ensure the continuity of their marriage and the welfare of their children. This means that women who do not justify wife-beating may be empowered and have a say in decision-making. Thus they are likely to turn down a man's desire towards having his ideal family size if the woman finds it unfavourable to her. Another study in sub-Saharan Africa has reported mixed findings where, in some countries, empowered women and those who did not justify wife-beating had more children than the ideal number. Such a finding was explained as more empowered women may be willing to comply with society's expectations of larger family sizes even though they may prefer a smaller family size.^18^

The present study also made interesting observations on predictors of the desired number of children. The study found that women who had an attitude towards justification of wife-beating and those who were involved in decision making had higher odds of having the ability to have the desired number of children. The reason could be that those who are involved in decision-making can negotiate with their spouses on the desired number of children.^20,25^ Yaya et al.^26^ also expounded on the value of children as a major driver for fertility preference. Although this study indicated women's autonomy as a protective factor for the desired number of children, evidence suggests a positive relationship between autonomy and contraception use providing a probable explanation for the former.^27–29^ Contrary to the findings of this study, no significant association was found between women's autonomy and the desired number of children in a study by Vlassoff.^30^ Collectively, this points to the potential importance of women's autonomy in reproductive-related decisions.

The study also found that women who were in the middle wealth index, whose husbands had a secondary education and who had a household size of 7–9 had the highest odds of having the ability to have the desired number of children. This finding agrees with the findings of Atake and Ali,^17^ where, as household socio-economic position and the husband's degree of education increase, so does the likelihood that a woman will have the ideal number of children.^17^ Women from big families are also more likely to have the desired number of children. This could be explained by the fact that in some nations, including Ghana, having several children is still seen as the finest insurance policy and the safest pension. Women who belonged to the Islamic religion had lower odds of having the ability to have the desired number of children. These are all consistent with several earlier investigations.^31–33^

The study also found that women who were the richest and whose husbands had higher education had the lowest odds of having the ideal number of children. Studies in China by Cao et al.^34^ and Zheng et al.^35^ reported similar findings. In Japan, Kim and Sung^36^ found an association between women's wealth status and the number of children they had. Specifically, they noted that women with higher socio-economic status tend to have fewer children. This finding also agrees with the findings of some western countries, including Canada, Iceland, Sweden, Norway and the USA.^37^ Studies conducted in other sub-Saharan African countries that reported similar findings, such as Obiyan et al. in Nigeria,^38^ are of more importance to the present study. In accordance with previous studies,^38–40^ the present study explains that wealthier women may be employed and have more negotiating power when it comes to fertility decisions. Such women may also adopt a variety of family planning methods to prevent unwanted pregnancies. The policy consequence for Ghana is that by elevating women's social standing, fertility strategies can achieve higher results.

The present study also found that women who had a household size of ≥10, belonged to the traditional religion and were in a polygamous union had the highest odds of having the ideal number of children. Significantly, in traditional African society, a premium is placed on having more children, and this has been confirmed by a study by Nyarko^41^ in northern Ghana, where having more children is understood as being wealthy, as it implies having more labour on the farm. Also, this association is likely to be explained by the negative attitude of practitioners of African traditional religion towards the use of contraception.^33^ The present study thus confirms the findings of previous studies that have identified an association between religious orientation and the ideal number of children.^31,32^ The finding on the relationship between polygamy and the ideal number of children is explained by Abdi et al.^42^ They noted that polygamy places a premium on procreation and co-wives tend to engage in competition for more children.

### Implications and recommendations for Ghana

The findings of this study carry significant implications for Ghana's health policies and interventions aimed at enhancing women's empowerment and fertility preferences. Based on the study's results, several recommendations can be suggested to the Ministry of Health and all stakeholders.

Given the observed negative association between women's justification of wife-beating and their ideal number of children, efforts to combat gender-based violence should be intensified. Education campaigns and community-based interventions can be implemented to challenge societal norms that condone violence against women. The Ministry of Health could collaborate with women's rights organizations to provide support services and legal assistance to survivors of gender-based violence.

Enhancing women's autonomy in decision-making regarding reproductive health is crucial. Policies and programs should focus on improving women's access to education, economic opportunities and resources. Empowering women to make informed choices about their reproductive health can contribute to achieving their desired number of children. The Ministry of Health can partner with educational institutions and community organizations to implement empowerment programs tailored to women's needs.

Given the association between socio-economic status and fertility preferences, poverty reduction and economic empowerment interventions should be prioritized. The government could implement income-generation initiatives and provide vocational training opportunities for women. Additionally, social safety nets and financial support programs targeting low-income households can help alleviate economic barriers to family planning.

Recognizing the impact of religious and cultural factors on fertility preferences, culturally sensitive approaches should be adopted in reproductive health programs. The Ministry of Health can collaborate with religious leaders and community elders to promote dialogue on family planning and reproductive rights within religious and cultural contexts. This could involve integrating family planning education into religious teachings and community gatherings.

Improving access to quality family planning services is essential for enabling women to realize their fertility preferences. The Ministry of Health should prioritize the expansion of reproductive health services, including contraceptive counselling, access to a variety of contraceptive methods and maternal healthcare. Efforts should also be made to address barriers such as geographic accessibility, cost and cultural stigma associated with family planning.

Continuous monitoring and evaluation of reproductive health programs are necessary to assess their effectiveness and identify areas for improvement. The Ministry of Health can invest in research initiatives to better understand the factors influencing women's fertility preferences and empowerment. Longitudinal studies and qualitative research can provide valuable insights into the complex dynamics shaping reproductive decision-making in Ghana.

By implementing these recommendations, Ghana can work towards promoting women's empowerment and reproductive health, ultimately contributing to the well-being of women and their families across the country.

### Strengths and limitations

Acknowledging the strengths and weaknesses inherent in the study design is essential. Significantly, this study used nationally representative data as well as quantitative methods. This means that the findings of the study are generalizable to all women in Ghana. Additionally, the study used higher-order statistical tools for the analysis. This ensured that the data were analysed rigorously to make the findings valid. However, since the study used a cross-sectional design, it is impossible to draw cause–effect inferences among the studied variables. In addition, recall and desirability biases are likely to affect the quality of the data used for the study. Finally, the ability of a woman to have the desired number of children can also be affected by other personal, family and societal variables that were not considered when calculating the empowerment index. These variables include people's attitudes towards abortion, inaccurate information about fertility, social ties, spatial mobility and traditional ideologies.^16^

## Conclusions

The present study examined the association between women's empowerment and the ideal number of children and their ability to have their desired number of children. We found that women's attitudes towards justification for wife-beating, autonomy, socio-economic status and religion were determinants of the ideal number of children. The study also found that women's attitude towards a justification for wife-beating, husband's educational attainment, wealth status and household size determined their ability to have the desired number of children. These findings present target areas for policies and interventions to check Ghanaian women's fertility preferences and enhance empowerment.

## Figures and Tables

**Figure:**
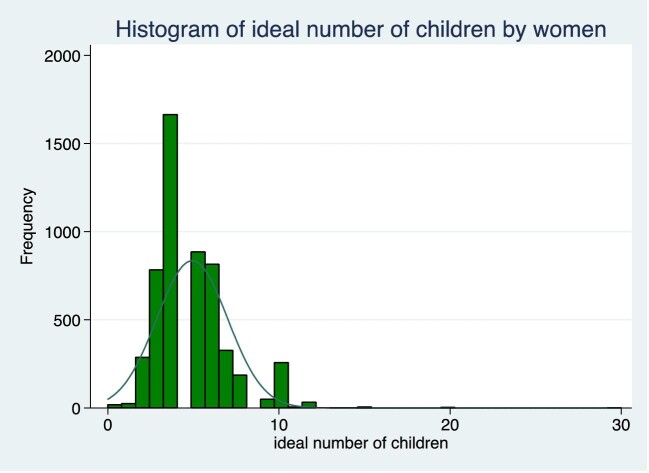
**Skewness/Kurtosis test**Pr(skewness) Pr(kurtosis)  0.000     0.000**Shapiro–Wilk test**W   V    Z  Prob>z0.92 242.18 14.43 0.000

## Data Availability

The datasets generated and/or analysed during this study are available in the DHS repository (https://dhsprogram.com).

## Appendix B

**Table:** Poisson regression of association of women’s empowerment and the ideal number of children

Variable	Model 1a, OR (95% CI)	Model 1b, aOR (95% CI)
Women’s empowerment		
Attitude towards violence score	0.92*** (0.91 to 0.94)	0.98** (0.96 to 0.99)
Autonomy score	0.89*** (0.88 to 0.90)	0.94*** (0.93 to 0.95)
Decision-making score	0.97*** (0.95 to 0.98)	1 (0.98 to 1.01)
Residence		
Urban		1 (1.00 to 1.00)
Rural		0.99 (0.95 to 1.02)
Wealth		
Poorest		1 (1.00 to 1.00)
Poorer		0.98 (0.94 to 1.02)
Middle		0.91*** (0.87 to 0.95)
Richer		0.88*** (0.84 to 0.93)
Richest		0.86*** (0.81 to 0.92)
Husband’s education		
None		1 (1.00 to 1.00)
Primary		0.92*** (0.88 to 0.96)
Secondary		0.87*** (0.84 to 0.90)
Higher		0.84*** (0.80 to 0.89)
Household size		
1–3		1 (1.00 to 1.00)
4–6		1.08*** (1.04 to 1.11)
7–9		1.16*** (1.11 to 1.21)
≥10		1.19*** (1.12 to 1.26)
Religion		
None		1 (1.00 to 1.00)
Christian		1.02 (0.95 to 1.10)
Islam		1.17*** (1.09 to 1.25)
Traditional		1.18*** (1.07 to 1.29)
Polygamous union		
No		1 (1.00 to 1.00)
Yes		1.04* (1.01 to 1.08)
N	5341	5341
Pseudo-R^2^	0.03	0.053

*p<0.05, **p<0.01, ***p<0.001.

## Appendix C

**Table:** Logistic regression of association of women empowerment and ability to desired number of children

Variable	Model 2a, OR (95% CI)	Model 2b, aOR (95% CI)
Women’s empowerment		
Attitude towards violence score	1.27*** (1.15 to 1.39)	1.25*** (1.13 to 1.39)
Autonomy score	0.75*** (0.69 to 0.81)	0.78*** (0.71 to 0.86)
Decision-making score	1.33*** (1.22 to 1.45)	1.31*** (1.19 to 1.44)
Residence		
Urban		1 (1.00 to 1.00)
Rural		0.84 (0.67 to 1.07)
Wealth		
Poorest		1 (1.00 to 1.00)
Poorer		1.33*(1.04 to 1.71)
Middle		1.43* (1.06 to 1.91)
Richer		1.24 (0.88 to 1.74)
Richest		1.11 (0.75 to 1.67)
Husband’s education		
None		1 (1.00 to 1.00)
Primary		1.09 (0.82 to 1.45)
Secondary		1.40** (1.09 to 1.80)
Higher		1.38 (0.97 to 1.96)
Household size		
1–3		1 (1.00 to 1.00)
4–6		4.49*** (3.48 to 5.78)
7–9		11.57*** (8.60 to 15.57)
≥10		8.52*** (5.72 to 12.69)
Religion		
None		1 (1.00 to 1.00)
Christian		1.3 (0.89 to 1.92)
Islam		0.55** (0.35 to 0.86)
Traditional		0.82 (0.47 to 1.41)
Polygamous union		
No		1 (1.00 to 1.00)
Yes		0.95 (0.74 to 1.21)
N	5341	5341
Pseudo-R^2^	0.026	0.121

*p<0.05, **p<0.01, ***p<0.001.
